# Allergic diseases of the skin and drug allergies – 2010. Intradermal skin testing with cefazolin regardless of the history of hypersensitivity to antibiotics

**DOI:** 10.1186/1939-4551-6-S1-P97

**Published:** 2013-04-23

**Authors:** Jae-Woo Kwon, Yoon-Jung Kim, Sae-Hoon Kim, Sang-Heon Cho, Kyung-up Min, Yoon-Seok Chang

**Affiliations:** 1 Division of Allergy and Clinical Immunology, Department of Internal Medicine, Kangwon National University College of Medicine, Chuncheon, South Korea; 2 Department of Internal Medicine, Seoul National University College of Medicine, Seoul, South Korea; 3 Internal Medicine, Seoul National University Bundang Hospital, Seongnam, South Korea

## Background

There have been no standard methods to predict the hypersensitivity to cephalosporin. The relationship between cephalosporin hypersensitivity and history of beta-lactam hypersensitivity is not clear. This retrospective study is to evaluate the reliability of routine prophylactic skin test with cefazolin in general population and the relationship between results of cefazolin skin testing and the history of beta-lactam hypersensitivity.

## Methods

The medical records of patientswho underwent skin testing to cefazolinfrom January 2010 to January 2011 at Bundang Seoul National University Hospital, South Korea were evaluated. Cefazolin was injected intradermaly with the concentration of 0.3 mg/ml without negative control. Skin testing to negative control was done for some of the patients who showed the positive results in cefazolin skin testing. History of beta-lactam hypersensitivity is taken from the statements of patients. Immediate adverse reactions after cefazolin injection were evaluated by searching key words including urticaria, itching, hypersensitivity, or anaphylaxis within 3 days after start of cefazolin in electronic chart and searching the consultations to allergy specialists or dermatologists after cefazolin injection. And then the medical records of searched patients were reviewed by an allergist.

## Results

There were 13,153 cases of skin testing with cefazolin during 13 months. Positive rate of cefazolin skin tests without negative and positive controls was 1.4%. Among 81 patients with history of suspicious beta-lactam hypersensitivity, 7 patients (9.9%) had positive results, as compared with 176 patients (1.3%) of patients without suchhistory (9.9% vs 1.3%, P < 0.0001). Among 19 patients who showed positive skin testing to cefazolin and then tested with negative control, 14 (73.4%) patients were proved as false positive with reactivity to normal saline. Among 1,152 patientsexamined for skin testing to cefazolin more than twice during 13 months, 21 patients (1.8%) showed different results in serial skin tests to cefazolin.

## Conclusions

This study suggests that routine prophylactic skin testing to cefazolin without negative control for all patients seems unreliable but prophylactic testing for patients with the history of beta-lactam hypersensitivity could be helpful, although the large prospective study is needed.

